# MicroRNAs and Autoimmune-Mediated Eye Diseases

**DOI:** 10.3389/fcell.2020.00818

**Published:** 2020-08-20

**Authors:** Yankai Wei, Na Li, Lu Zhao, Chao Yang, Binyun Ma, Xiaorong Li, Ruihua Wei, Hong Nian

**Affiliations:** ^1^Tianjin Key Laboratory of Retinal Functions and Diseases, Tianjin International Joint Research and Development Centre of Ophthalmology and Vision Science, Eye Institute and School of Optometry, Tianjin Medical University Eye Hospital, Tianjin, China; ^2^Department of Medicine/Hematology, Keck School of Medicine of the University of Southern California, Los Angeles, CA, United States

**Keywords:** miRNAs, autoimmune uveitis, Grave’s ophthalmopathy, Sjögren’s syndrome dry eye, biomarkers, therapeutic targets

## Abstract

MicroRNAs (miRNAs) are evolutionarily conserved short non-coding RNAs that act at post-transcriptional regulation of gene expression by destroying target messenger RNA or inhibiting its translation. Recently, miRNAs have been identified as important regulators in autoimmunity. Aberrant expression and function of miRNAs can lead to dysfunction of immune system and mediate autoimmune disorders. Here, we summarize the roles of miRNAs that have been implicated in three representative ocular autoimmune disorders, including autoimmune uveitis, Grave’s ophthalmopathy, and Sjögren’s syndrome dry eye, and discuss the potential of miRNAs as biomarkers and therapeutic targets for the diagnosis and treatment of these diseases.

## Introduction

MicroRNAs (miRNAs), a class of evolutionarily conserved, short non-coding RNAs, are potent post-transcriptional regulators of gene expression through binding to the 3’ untranslated region of a target mRNA (Bartel, 2004). Since the discovery of the first miRNA in *Caenorhabditis elegans* in 1993, miRNAs have been identified as key players in a plethora of cellular processes, such as cell growth, proliferation, differentiation, and apoptosis (Lee et al., 1993; Ambros, 2004). Besides, miRNAs are critical for normal development and function of immune system, and abnormal expression and function of miRNAs can lead to immunological aberrations and autoimmunity (Mehta and Baltimore, 2016). Recently, accumulating evidence from animal models and clinical studies has revealed the critical significance of miRNAs in the pathogenesis of many autoimmune diseases (Chen et al., 2016; Garo and Murugaiyan, 2016; Long et al., 2018), including autoimmune-mediated eye diseases.

**FIGURE 1:**
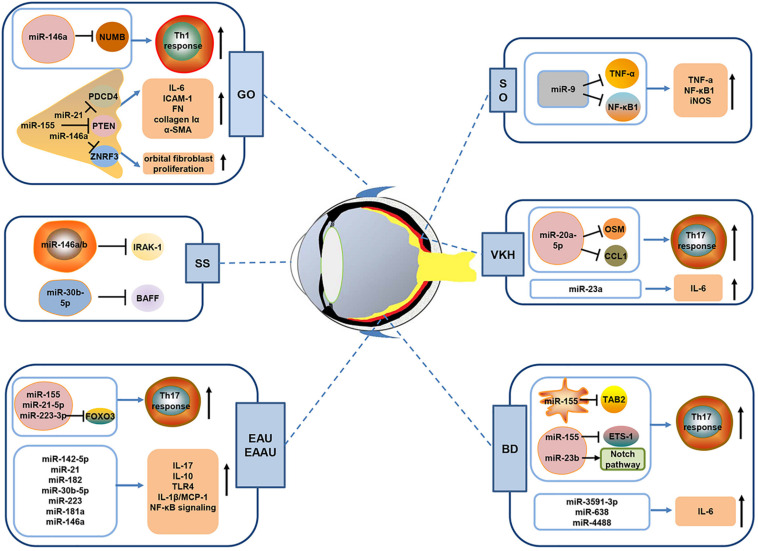
Schematic diagram showing the possible mechanisms of miRNAs in autoimmune-mediated eye diseases. A growing body of evidence has shown that miRNAs are implicated in the pathogenesis of autoimmune-mediated eye diseases including autoimmune uveitis (EAU, EAAU, BD, VKH and SO), Grave’s ophthalmopathy (GO), and Sjögren’s syndrome (SS) dry eye. As shown in Figure, dysregulated miRNAs can promote the production of pro-inflammatory cytokine and enhance the activity of inflammatory pathways in autoimmune uveitis. Besides, some miRNAs (including miR-223-3p, miR-155, miR-23b, and miR-20a-5p) have been demonstrated to promote Th17 cells response via suppression of their target gene expression. In GO, aberrantly expressed miRNA, such as miR-146a, miR-155, and miR-21, could affect Th1 cell response or orbital fibroblasts proliferation to regulate disease development. In addition, miR-146a/b and miR-30b-5p in SS patients may negatively regulate IRAK-1 and BAFF, which are key molecules that initiate SS development. EAU, experimental autoimmune uveitis; EAAU, experimental autoimmune anterior uveitis; BD, Behcet’s disease; VKH, Vogt–Koyanagi–Harada; SO, sympathetic ophthalmia.

The eye, an immune privileged organ, has adapted several negative regulators to prevent inflammation within its tissue microenvironment (Benhar et al., 2012; de Andrade et al., 2016). These regulators suppress inflammatory activity, induce production of ant-inflammatory cytokines and mediate the activation of tolerogenic antigen-presenting cells and regulatory T cells (Taylor, 2016; Keino et al., 2018). However, in the inflammatory setting, aberrant activation of effector immune cells (e.g., T cells and B cells) and excessive expression of proinflammatory mediators contribute to the breakdown of ocular immune privilege and elicit the development of autoimmunity (Caspi, 2006; Stern et al., 2010). Here, we summarize the current knowledge on miRNAs dysregulation (Table 1) and their pathogenic roles in three autoimmune-mediated eye diseases, including autoimmune uveitis, Grave’s ophthalmopathy, and Sjögren’s syndrome dry eye (Figure 1), focusing on three aspects: (1) alterations in miRNAs expression profiles, (2) the effect of aberrant miRNA expression on the onset and progression of diseases, and (3) the related molecular mechanisms. Moreover, we discuss the potential of miRNAs as potent biomarkers and therapeutic targets for the diagnosis and treatment of these diseases.

**TABLE 1:** Differential expression of miRNAs in ocular autoimmune disorders.

Disease	Sample	Upregulated miRNA	Downregulated miRNA	Target	References
**Experimental autoimmune uveitis**	Retina	miR-223	miR-181a		Watanabe et al., 2016
		miR-146a			
	Spleen, lymph nodes and eye tissues		miR-30b-5p	IL-10; TLR4	Sun et al., 2018
	Ocular tissues	miR-142-5p	miR-182		Ishida et al., 2011
		miR-21			
	CD4 + T cells	miR-155			Escobar et al., 2013
	Retina and splenic lymphocytes	miR-21-5p		IL-10	Shi et al., 2019
	Th17 cells	miR-223-3p		FOXO3	Wei et al., 2019
**Experimental autoimmune anterior uveitis**	Iris/ciliary bodies; popliteal lymph node	miR-9-3p	miR-146a-5p		Hsu et al., 2015
		miR-182-5p	miR-155-5p		
		miR-183-5p	miR-147b		
			miR-223-3p		
**Behcet’s disease**	PBMCs	miR-155			Kolahi et al., 2018
	PBMCs	miR-3591-3p	miR-638		Woo et al., 2016
			miR-4488		
	PBMCs	miR-326	miR-21		Jadideslam et al., 2019
	PBMCs and dendritic cells		miR-155	TAB2	Zhou et al., 2012
	CD4 + T cells	miR-155		Ets-1	Na et al., 2016
	CD4 + T cells		miR-23b		Qi et al., 2014
	Serum	miR-146a			Ibrahim et al., 2019
**Vogt–Koyanagi–Harada syndrome**	CD4 + T cells		miR-20a-5p	OSM; CCL1	Chang et al., 2018
**Sympathetic ophthalmia**	globes		miR-9	TNF-α; NF-κB1	Kaneko et al., 2012
			miR-let-7e		
			miR-1		
			miR-182		
**Grave’s ophthalmopathy**	Orbital adipose tissue	miR-146a			Jang et al., 2016, 2018
	CD4 + T cells		miR-146a	NUMB	Hu et al., 2017; Yang et al., 2017
	Orbital fibroblast	miR-146a		ZNRF3	Woeller et al., 2019
		miR-155		PTEN	
	Orbital fibroblast	miR-21			Tong et al., 2015
	Orbital fibroblast	miR-21		PDCD4	Lee et al., 2016
	Orbital fat tissue		miR-27a		Jang et al., 2019
			miR-27b		
	CD4 + T cells	miR-183		EGR-1	Thiel et al., 2019
		miR-96			
	Serum		miR-146a		Wei et al., 2014
**Sjögren’s syndrome dry eye**	Tears	miR-16-5p	miR-30b-5p		Kim et al., 2019
		miR-34a-5p	miR-30c-5p		
		miR-142-3p	miR-30d-5p		
		miR-223-3p	miR-92a-3p		
			miR-134-5p		
			miR-137		
			miR-302d-5p		
			miR-365b-3p		
			miR-374c-5p		
			miR-487b-3p		
	PBMCs		miR-150-5p		Chen et al., 2017a
	CD4 + T cell	miR-155-5p	miR-let-7d-3p		Wang-Renault et al., 2018
		miR-222-3p	miR-30c-5p		
		miR-146a-5p	miR-378a-3p		
		miR-28-5p			
	CD19 + B cell		miR-30b-5p	BAFF	
		miR-222-3p	miR-378a-3p		
			miR-26a-5p		
			miR-19b-3p		
	PBMCs	miR-146a			Pauley et al., 2011; Shi et al., 2014
	PBMCs	miR-146a/b		IRAK-1	Zilahi et al., 2012
	PBMCs		miR-155		Shi et al., 2014
	PBMCs	miR-155			Pauley et al., 2011; Chen et al., 2017b
	PBMCs	miR-181a			Peng et al., 2014

## MicroRNAs and Different Types of Uveitis

### MicroRNAs and Experimental Autoimmune Uveitis

Autoimmune uveitis is an intraocular inflammatory disease characterized by immune-mediated damage in the uveal tissues and retina. The progressive irreversible photoreceptors’ damage caused by autoreactive T cells eventually leads to visual impairment and even blindness (Caspi, 2010). Experimental autoimmune uveitis (EAU) is an animal model of human uveitis, and its etiology has not been fully discerned (Caspi et al., 2008).

Dysregulation of certain miRNAs has been shown to be closely associated with the progression of EAU. Upregulation of miR-223 and miR-146a and downregulation of miR-181a were observed in the retina of rats during the course of EAU, corresponding with the score of EAU and the upregulation of IL-1β/MCP-1 (Watanabe et al., 2016). Additionally, 36 upregulated miRNAs and 31 downregulated miRNAs were found in peripheral blood lymphocytes from rats with EAU (Guo et al., 2015), and targets of these differentially expressed miRNAs were implicated in some immune signaling pathways such as T-cell receptor signaling pathway and Toll-like receptor signaling pathway, both of which are closely associated with the pathogenesis of EAU (Horai et al., 2013; Xiao et al., 2016). More recently, it was shown that miR-30b-5p expression was decreased in spleen, lymph nodes, and eye tissues of EAU rats, and miR-30b-5p directly targeted IL-10 and TLR4 in T cells and reduced the level of IL-10 and TLR4 positive cells, regulating the development of EAU (Sun et al., 2018).

Th17 cells driven by IL-23 are central to the pathogenesis of EAU (Peng et al., 2007). Th17 cell program is tightly regulated by various transcription factor, such as RORγt, STAT3, and FOXO3 (Yang et al., 2007; Maddur et al., 2012; Wei et al., 2019). IL-17, the signature cytokine of Th17 cells, has been shown to promote inflammation and tissue damage (Sun et al., 2015). Recent advances have revealed the important role of miRNAs in regulating Th17 cell response. Ishida et al. identified the increased expression of miR-142-5p and miR-21 and reduced expression of miR-182 in ocular tissues from mice with EAU, which paralleled the dynamic expression of IL-17. It was implied that these dysregulated miRNAs might regulate the development of EAU by affecting the expression of IL-17 (Ishida et al., 2011). Moreover, it has been reported that STAT3 binds directly to the miR-155 locus and induces its expression, and STAT3/miR-155 axis promotes the expansion of pathogenic Th17 cells, thereby contributing to the development of EAU (Escobar et al., 2013). More recently, it was revealed that miR-21-5p might affect EAU progression by altering Th17/Treg balance (Shi et al., 2019). Our own group has verified that miR-223-3p was significantly upregulated in IRBP-specific Th17 cells, and knockdown of miR-223-3p decreased the pathogenicity of Th17 cells in a T-cell transfer model of EAU. Mechanistic studies showed that miR-223-3p directly repressed the expression of FOXO3, and FOXO3 negatively regulated pathogenic Th17 responses partially via suppression of IL-23R expression (Wei et al., 2019). Collectively, these observations indicate that miRNAs are vital regulators of Th17 responses and provide new insights into the molecular pathogenesis of EAU.

### MicroRNAs and Experimental Autoimmune Anterior Uveitis

Experimental autoimmune anterior uveitis (EAAU), in which inflammation is restricted to ocular anterior segment while retinal tissues and photoreceptor cells are not involved, resembles human idiopathic anterior uveitis (Broekhuyse et al., 1991). EAAU is characterized histologically by the infiltration of lymphocytic and mononuclear cell in the anterior uvea. The etiologies are largely unknown, but epigenetic mechanisms are paving the way for a better understanding of this disease. Recently, Hsu et al. (2015) demonstrated that the expression of miR-146a-5p, miR-155-5p, miR-147b, and miR-223-3p was decreased after EAAU induction, whereas the expression of miR-9-3p, miR-182-5p, and miR-183-5p was elevated. In addition, both the secretion of IFN-γ, IL-17, IL-12A, IL-1β, and IL-6 in aqueous humor and their mRNA expression in iris and ciliary bodies were upregulated in rats with EAAU as compared to controls. Based on these observations, it was speculated these differentially expressed miRNAs might promote Th1 and Th17 specific cytokine production, thereby contributing to the pathogenesis of EAAU, but the potential roles of miRNAs in the EAAU are still in the early discovery stage and need to be fully explored in the future.

### MicroRNAs and Behcet’s Disease

Behcet’s disease (BD) is a systemic inflammatory disease of unknown etiology, characterized by ocular lesions, oral ulcer, genital ulcer, and multiple skin lesions (Yang et al., 2008; Zeidan et al., 2016). The eye involvement mainly manifests with chronic, recurrent bilateral non-granulomatous uveitis with necrotizing retinal vasculitis (Park et al., 2014). The pathogenesis of BD is highly complex, with immunological aberrations, environmental factors, genetic predisposition, and epigenetic alterations involved, but remains largely unknown (Greco et al., 2018). In recent years, studies have paid much attention to the critical implication of miRNAs in the development of BD.

The disturbed miRNA expression in peripheral blood mononuclear cells (PBMCs) from patients with BD has been unveiled, and certain miRNAs have been deemed as potent diagnostic biomarkers. MiR-155 expression was significantly increased in the PBMCs of BD patients with uveitis, whereas miR-146a expression had no significant difference between BD patients and controls (Kolahi et al., 2018). In addition, altered expression of miR-638, miR-4488, and miR-3591-3p in the PBMCs of BD patients was also reported (Woo et al., 2016), which is associated with the production of IL-6, an inflammatory cytokine involved in the pathogenesis of BD (Lin, 2015). More recently, Jadideslam and colleagues compared the expression of miR-21, miR-146b, and miR-326 in PBMCs from Iranian Azari BD patients with that in healthy controls, and revealed the downregulation of miR-21 and upregulation of miR-326. Furthermore, they suggested the great potential of miR-326 as a biomarker for predicting the uveitis and severe eye involvement in BD (Jadideslam et al., 2019).

Th17 cells can facilitate neutrophil inflammatory response that underlies the pathogenesis of BD (Leccese and Alpsoy, 2019). Increased frequencies of Th17 cells have been reported in BD patients with active uveitis (Chi et al., 2008). Recent evidence from clinical studies implicates miRNAs as key regulators of Th17 responses in BD. Zhou et al. (2012) found that miR-155 inhibited dendritic cell-driven Th17 responses by targeting TGF-beta-activated kinase 1 binding protein 2 (TAB2), while Na et al. (2016) demonstrated upregulated miR-155 in CD4+ T cells promoted Th17 responses via suppression of E26 transformation-specific-1 (Ets-1) in BD. The difference in cells types and disease stage may account for the discrepancy in these studies. Besides, Qi et al. (2014) revealed that downregulation of miR-23b was closely associated with activation of the Notch pathway and expansion of Th1/Th17 cells, hinting the important role of miR-23b in the pathogenesis of BD.

Genome-wide association studies have identified that single nucleotide polymorphisms (SNPs) in miRNAs are involved in many autoimmune diseases, including BD. For example, there is a strong correlation between rs2910164 in the pre-miR-146a gene and development of BD (Zhou et al., 2014; Oner et al., 2015; Ibrahim et al., 2019). Decreased miR-146a expression, as well as reduced proinflammatory cytokine production (including IL-17, TNF-α, and IL-1β), was found in individuals carrying rs2910164 CC genotype and C allele. It was implied that this SNP might protect against BD possibly via suppression of miR-146a expression and proinflammatory cytokine production. Moreover, miR-196a2/rs11614913 confers susceptibility to BD by regulating miR-196a expression and proinflammatory cytokine (IL-1β and MCP-1) secretion (Qi et al., 2013). Another extensively investigated variant involved in autoimmune diseases is the pre-miR-499 rs3746444 polymorphism. This SNP has been shown to be associated with an increased risk of BD in a Turkish population (Oner et al., 2015). In addition, reduced CC genotype and C allele frequencies of miR-182/rs76481776 in BD patients were also revealed (Yu et al., 2014).

MiRNAs and their SNPs affect cells and molecules involved in BD, which deepens our understanding of BD pathogenesis and sheds new light on the diagnosis and treatment of BD. More in-depth studies are warranted to explore the miRNAs-mediated mechanisms in the development of BD.

### MicroRNAs and Vogt–Koyanagi–Harada Syndrome

Vogt–Koyanagi–Harada (VKH) syndrome, one of the sight-threatening diseases, is characterized by bilateral granulomatous uveitis associated with neurological, auditory, and dermatological manifestations, which are presumably caused by T-cell-mediated autoimmune response against melanocyte-associated antigens in multiple organs (Du et al., 2016; Silpa-Archa et al., 2016). Active VKH patients express enhanced Th17 cell responses (Yang et al., 2013). Most recently, Chang et al. reported that the expression of miR-20a-5p was decreased in the CD4 + T cells of active VKH patients, and miR-20a-5p negatively regulated IL-17 production via suppression of the expression of oncostatinM (OSM) and C–C motif chemokine ligand 1 (CCL1) as well as the activity of the PI3K-AKT pathway (Chang et al., 2018), highlighting the involvement of miR-20a-5p in Th17 cell responses during VKH. Moreover, copy number variations of miR-23a, miR-146a, and miR-301a as well as genetic variants of miR-182 have been revealed to confer risk for VKH disease (Yu et al., 2014; Hou et al., 2016). Further functional studies indicated that miR-23a might contribute to the development of VKH syndrome by promoting the production of IL-6 (Hou et al., 2016). miRNA research in the VKH syndrome remains in its infancy, and more profound research is warranted and will facilitate the delineation of novel diagnostic biomarkers and therapeutic targets in VKH.

### MicroRNAs and Sympathetic Ophthalmia

Sympathetic ophthalmia (SO) is a rare, granulomatous uveitis found in bilateral eyes, occurring after ocular trauma and intraocular surgery. It is mainly characterized by acute or chronic uveitis, accompanied by mutton-fat KP in anterior segments and yellow-white lesions of choroid in the posterior segment (Damico et al., 2005; Chang and Young, 2011). The etiology is not clearly understood, but a vast majority of studies indicate that T-cell-mediated autoimmune responses against ocular self-antigens is critically implicated in the pathogenesis of SO (Chan and Mochizuki, 1999).

Although the accumulating data have demonstrated the crucial roles of miRNAs in immune cell function, the role of miRNAs in SO pathogenesis is still largely unknown and only one research group reported this topic. It was shown that four miRNAs including miR-1, let-7e, miR-9, and miR-182, which are associated with T-cell-mediated inflammatory pathway, were downregulated in globes of patients with SO compared to those in controls. Among them, hsa-miR-9 directly targeted proinflammatory TNF-α and NF-κB1, both of which are crucial factors in the pathogenesis of SO (Kaneko et al., 2012). Therefore, the specific role of miRNAs in SO is calling for extensive research.

## MicroRNAs and Grave’s Ophthalmopathy

Grave’s ophthalmopathy (GO), an extra-thyroidal complication of Graves’ disease, is characterized by the inflammation and extensive remodeling of orbital adipose/connective tissues. Its clinical manifestations include exophthalmos, eyelid retraction, strabismus, and exposure keratitis, which may cause cosmetic and functional deficits (Dik et al., 2016). Although the precise pathophysiology of GO remains unclear, increasing evidence has shown this disease may result from the autoimmune reactions in which the sensitive T cells as well as autoantibodies against common antigens [including thyrotropin receptor (TSHR), insulin-like growth factor-1 receptor, thyroglobulin, calsequestrin (CASQ1) and collagen XIII] contribute to the activation and proliferation of orbital fibroblasts (OFs), resulting in extraocular tissues edema and fibrosis (Lahooti et al., 2010; Bahn, 2015; Shanmuganathan et al., 2015; Lacheta et al., 2019). Recently, particular attention has been paid to the role of miRNAs in the pathogenesis of GO.

MiR-146a is a key regulator of orbital tissue fibrosis and GO development. Upregulation of miR-146a in OFs suppressed the production of inflammatory protein [including IL-6 and intercellular adhesion molecule-1 (ICAM-1)] and TGF-β-induced fibrotic markers [fibronectin (FN), collagen Iα and α-smooth muscle actin protein (α-SMA)], suggesting a key role for miR-146a in anti-inflammatory and anti-fibrotic process (Jang et al., 2016, 2018). Conversely, downregulation of miR-146a in CD4 + T cells has been shown to contribute to the development of GO by promoting pro-inflammatory Th1 cytokine production and human T cells proliferation or via targeting NUMB (Hu et al., 2017; Yang et al., 2017). Most recently, Collynn and colleagues revealed that the TSHR signaling in GO patients can enhance OFs proliferation partially via induction of miR-146a and miR-155, and the effects of miR-146a and miR-155 may be due to their suppression on zinc and ring finger 3 (ZNRF3) and phosphatase and tensin homolog (PTEN) that normally limit cell proliferation (Woeller et al., 2019).

In addition to miR-146a, miR-21 also plays a pivotal role in the regulation of fibrosis. Tong et al. (2015) demonstrated that the expression of miR-21 was increased in OFs from patients with GO compared to that in control group. MiR-21 promoted OF proliferation and differentiation and suppressed the apoptosis, which contribute to the fibrosis of extraocular muscles. Moreover, another study revealed that platelet-derived growth factor-BB increased the expression level of miR-21 in OFs, and miR-21 mediated platelet-derived growth factor-BB induced downregulation of programed cell death 4 (PDCD4) in OFs, thereby contributing to cell proliferation and GO development (Lee et al., 2016).

With regard to other miRNAs, decreased miR-27a and miR-27b have been observed in orbital fat tissue from patients with GO. The overexpression of miR-27a and miR-27b leads to a significant reduction in the expression of adipogenesis-related genes such as PPARγ, C/EBPα, and C/EBPβ, suggesting a possible role of miR-27a and miR-27b in adipocyte development (Jang et al., 2019). Additionally, a recent study found that miR-183 and miR-96 were upregulated in CD4 + T cells from peripheral blood of GO patients. miR-183 and miR-96 targeted early growth response protein 1 (EGR-1) to regulate PTEN/Akt signaling, contributing to the activation of CD4 + T cells (Thiel et al., 2019). Besides, let-7b, which was upregulated in PBMCs, serum, and thyroid tissue of patients with Graves’ disease, was verified to directly suppress promyelocytic leukemia zinc finger (PLZF) expression and enhance the expression of TSHR in thyroid cells *in vitro* (Chen et al., 2018). Further studies including primary human orbital tissue or animal models are needed to determine the interaction of let-7b and TSHR signaling in GO.

Recently, investigators have linked polymorphisms in thyroid-specific and immune-modulating genes to the susceptibility to GO, which open novel avenues on understanding this disease. For example, Beata et al. found that rs179247 TSHR polymorphism was correlated with lower risk of GO in young GD patients (Jurecka-Lubieniecka et al., 2014). Moreover, the results from Lahooti et al. revealed an association between the CASQ1 SNP rs74123279, rs3838216, and rs2275703 and the development of GO (Lahooti et al., 2015). However, the roles of SNPs in miRNA genes in GO remain unexplored, awaiting further investigation.

## MicroRNAs and Sjögren’s Syndrome Dry Eye

Sjögren’s syndrome (SS) is a chronic systemic autoimmune disease, mainly characterized by lymphocytic infiltration of lacrimal and salivary glands, which results in ocular and oral dryness (de Paiva and Rocha, 2015). The pathogenesis of SS is multi-facetted and largely unknown. It has been reported that activation of innate and adaptive immune pathways, including type I IFN pathway, TGF-β/SMAD/Snail signaling pathway, and B cell activating factor (BAFF)/BAFF receptor axis, plays a crucial role in the pathogenesis of SS (Mavragani, 2017; Sisto et al., 2018). Recently, studies have established a close relationship between miRNAs dysregulation and the pathogenesis of SS.

The altered miRNA expression in immune cells from patients with SS was recently reported. Chen et al. (2017a) indicated 26 miRNAs with aberrant expression pattern in PBMCs from primary Sjögren’s syndrome (pSS) patients. Among them, the downregulation of miR-150-5p is a novel finding. In addition, Wang-Renault et al. found that in CD4 + T cells from patients with pSS, miR-let-7d-3p, miR-30c-5p, and miR-378a-3p were significantly downregulated, while miR-155-5p, miR-222-3p, miR-146a-5p, and miR-28-5p were upregulated. In CD19 + B cells, the expression of miR-378a-3p, miR-26a-5p, miR-30b-5p, and miR-19b-3p was reduced in pSS, while miR-222-3p expression was increased (Wang-Renault et al., 2018). Of note, miR-30b-5p was further identified as a negative regulator of BAFF, one of the key molecules that initiate SS development (Nocturne and Mariette, 2013). Moreover, a SS-specific miRNA profile in CD14 + monocytes was also displayed (Williams et al., 2016). Determining the functional contribution of these miRNAs to SS may clarify previously unknown cellular processes and unveil new potential therapeutic targets.

The dysregulation of miR-146a may be associated with the pathogenesis of SS. A previous study reported increased miR-146a expression in PBMCs from both SS patients and Sjs-prone mouse, which may affect innate immunity and contribute to the initiation and progression of SS (Pauley et al., 2011). In addition, Zilahi et al. (2012) found that miR-146a/b expression was upregulated in PBMCs from SS patients compared to that in healthy controls, while IL-1 receptor-associated kinase 1 (IRAK1) expression was downregulated, implying the existence of transcriptional repression of IRAK1 by miR-146a in SS patients. Another study verified the over-expression of miR-146a in PBMCs from SS patients and demonstrated that there was a positive correlation between the expression level of miR-146a and the VAS scores for dry eyes (Shi et al., 2014). All these studies suggest that upregulation of miR-146a may contribute to the development of SS, albeit the underlying mechanism need to be further determined.

Regarding other miRNAs, Shi et al. (2014) found that the expression of miR-155 was reduced in PBMCs from untreated Asian pSS patients. On the contrary, another two studies found that the expression of miR-155 was elevated in PBMCs from European and American pSS patients (Pauley et al., 2011; Chen et al., 2017b). The opposite findings may be due to different inclusion criteria for patients and distinct sample size applied in the studies. Additionally, Peng et al. (2014) revealed the upregulation of miR-181a and multiple virus-derived miRNAs in PBMCs from Chinese patients with pSS, indicating the possible role of miR-181a and virus infection in pSS. Therefore, the changes of miRNA expression in pSS patients indicate the potential clinical implications of miRNAs in this disease. However, the underlying mechanisms that regulate miRNA expression and the roles of deregulated miRNAs in SS pathogenesis remain to be defined.

## Circulating MicroRNAs as Biomarkers for Autoimmune-Mediated Eye Diseases

The potential value of miRNAs as biomarkers has gained tremendous interests in recent years. The disease-specific expression pattern makes some of them suitable biomarker candidates. Owing to their encapsulation in extracellular vesicles (EVs) or association with RNA-binding proteins, miRNAs found in various body fluids are highly stable (Schwarzenbach et al., 2014). Moreover, they are easily measured with the advances in detection technology such as microarray and deep sequencing (Schwarzenbach et al., 2014). It has been found that some miRNAs present in biofluids exhibit altered levels in ocular autoimmune disorders. As evidenced by Wei et al. (2014), miR-146a was downregulated in the serum of patients with active GO, with a negative correlation between the miR-146a level and clinical activity score. Ibrahim et al. (2019) found that elevated serum levels of miR-146a were closely associated with eye activity of BD patients, implying its diagnostic value. The discrepancy in the expression patterns of serum miR-146a in active GO and BD patients may be attributed to the complex mechanisms of the different ocular autoimmune diseases. In addition to serum, tear is also applicable as a source for circulating miRNAs. Recently, Kim et al. (2019) revealed four miRNAs being upregulated and 10 miRNAs being downregulated in tear samples of SS patients, indicating that tear miRNAs may provide clues to the pathogenesis of lacrimal gland dysfunction in SS patients. Hence, it seems that finding of new circulating miRNAs shows significant promise for the diagnosis or understanding of biology processes in autoimmune-mediated eye diseases. As a variety of issue, with respect to miRNAs, are addressed, circulating miRNAs may be applied as non-invasive biomarkers for clinical practice in the near future (Zhang et al., 2020).

## MicroRNAs as Therapeutic Targets for Autoimmune-Mediated Eye Diseases

MicroRNAs are emerging as potential molecular targets for the treatment of a variety of diseases owing to their unique expression profiles, crucial regulatory functions, and target specificity (Lu et al., 2019). Currently, in the cancer, heart disease and diabetes field, miRNA-based therapeutics have entered clinical trials stage (Li and Rana, 2014; Takahashi et al., 2019). To use miRNAs as therapeutic agents for ocular autoimmune diseases, it is required to maintain their stability and deliver them to ocular tissues efficiently. Adenovirus-mediated gene expression has efficiently transduced foreign genes into ocular tissues (Mallam et al., 2004), making adenovirus vectors attractive for delivery of miRNAs in the treatment of uveitis. A recent study by Shi et al. (2019) found that subretinal injection of anti-miR-21-5p adenovirus alleviated retinal injury and apoptosis in EAU mice by increasing IL-10 and decreasing IL-17, TNF-α and IFN-γ production. Moreover, Hsu et al. (2017) showed that administration of locked nucleic acid miR-146a mimics via intravitreal injection effectively dampened intraocular inflammation in EAAU. The therapeutic effects may partly be ascribed to increased miR-146a mimics stability by locked nucleic acid modification and good intraocular concentration provided by intravitreal injection. More recently, exosomes have been shown to be potential therapeutics through RNA transfer mechanisms (Tran et al., 2019), leading to their emergence as promising vehicles for delivering therapeutic miRNAs. Studies have demonstrated that mesenchymal stem cell (MSC)-derived exosomes could be manipulated to deliver miRNAs to exhibit their therapeutic potential (Che et al., 2019; Lou et al., 2020). However, the research on MSC-derived exosomes as carriers of miRNAs to treat autoimmune-mediated eye diseases remains in the early stage, calling for more extensive investigations.

## Conclusion

MicroRNA alterations are closely associated with the pathogenesis of ocular autoimmune disorders. Due to the important roles of miRNAs in regulating inflammation and immune response, miRNAs can be potentially therapeutic targets for autoimmune-mediated eye diseases. Nevertheless, the exact roles of most miRNAs and the underlying mechanisms have not been clarified, requiring further investigation. Additionally, for successful translation to clinical therapies, it is necessary to develop safe and effective delivery system that can transport therapeutic miRNAs specifically to target sites.

## Author Contributions

YW read the literature related to the topic and participated in drafting the manuscript. NL, LZ, and CY participated in searching and archiving the literature related to the topic and discussed the contents of the manuscript. BM, XL, and RW revised the manuscript. HN participated in the design, revision, and final approval of the manuscript. All authors read and approved the final manuscript.

## Conflict of Interest

The authors declare that the research was conducted in the absence of any commercial or financial relationships that could be construed as a potential conflict of interest.

## Abbreviations

miRNAs: microRNAs
EAU: experimental autoimmune uveitis
EAAU: experimental autoimmune anterior uveitis
BD: Behcet’s disease
PBMCs: peripheral blood mononuclear cells
TAB2: TGF-beta activated kinase 1 binding protein 2
Ets-1: E26 transformation specific-1
SNP: single nucleotide polymorphism
VKH: Vogt–Koyanagi–Harada
OSM: oncostatin M
CCL1: C–C motif chemokine ligand 1
SO: sympathetic ophthalmia
GO: Grave’s ophthalmopathy
CASQ1: calsequestrin
OF: orbital fibroblast
ICAM-1: intercellular adhesion molecule-1
FN: fibronectin
α-SMA: α-smooth muscle actin protein
ZNRF3: zinc and ring finger 3
PTEN: phosphatase and tensin homolog
PDCD4: programed cell death 4
PLZF: promyelocytic leukemia zinc finger
SS: Sjögren’s syndrome
BAFF: B-cell activating factor
pSS: primary Sjögren’s syndrome
IRAK1: IL-1 receptor-associated kinase 1
MSC: mesenchymal stem cell.
